# Geographic distributions of *Idh*-1 alleles in a cricket are linked to differential enzyme kinetic performance across thermal environments

**DOI:** 10.1186/1471-2148-9-113

**Published:** 2009-05-21

**Authors:** Diana L Huestis, Brenda Oppert, Jeremy L Marshall

**Affiliations:** 1 Department of Entomology, Kansas State University, Manhattan, KS 66506, USA; 2 Laboratory of Malaria and Vector Research, National Institute of Allergy and Infectious Diseases, National Institutes of Health, 12735 Twinbrook Parkway, Rockville, MD 20852, USA; 3 USDA ARS Grain Marketing and Production Research Center, Manhattan, KS 66502, USA

## Abstract

**Background:**

Geographic clines within species are often interpreted as evidence of adaptation to varying environmental conditions. However, clines can also result from genetic drift, and these competing hypotheses must therefore be tested empirically. The striped ground cricket, *Allonemobius socius*, is widely-distributed in the eastern United States, and clines have been documented in both life-history traits and genetic alleles. One clinally-distributed locus, isocitrate dehydrogenase (*Idh*-1), has been shown previously to exhibit significant correlations between allele frequencies and environmental conditions (temperature and rainfall). Further, an empirical study revealed a significant genotype-by-environmental interaction (GxE) between *Idh*-1 genotype and temperature which affected fitness. Here, we use enzyme kinetics to further explore GxE between *Idh*-1 genotype and temperature, and test the predictions of kinetic activity expected under drift or selection.

**Results:**

We found significant GxE between temperature and three enzyme kinetic parameters, providing further evidence that the natural distributions of *Idh*-1 allele frequencies in *A. socius *are maintained by natural selection. Differences in enzyme kinetic activity across temperatures also mirror many of the geographic patterns observed in allele frequencies.

**Conclusion:**

This study further supports the hypothesis that the natural distribution of *Idh*-1 alleles in *A. socius *is driven by natural selection on differential enzymatic performance. This example is one of several which clearly document a functional basis for both the maintenance of common alleles and observed clines in allele frequencies, and provides further evidence for the non-neutrality of some allozyme alleles.

## Background

Individuals within populations are under selection pressure to adapt to their environment; these adaptations can be morphological, physiological, or behavioral in nature. However, these diverse adaptations all have a molecular basis and the study of molecular adaptation to environmental conditions is an active area of research within evolutionary biology. One biochemical adaptation that lends itself to empirical study is the kinetic performance of different enzyme alleles (allozymes) under a range of environmental conditions, such as temperature. Allozyme alleles arise when a point mutation in the protein-coding sequence leads to an amino acid substitution which alters the charge, weight, and folding of the protein [1]. Amino acid substitutions may also affect the function of the protein, altering optimal ranges for temperature, pH, or substrate concentration. Most amino acid changes will likely be deleterious and quickly eliminated by purifying selection [2,3]. However, some substitutions may not significantly affect the function of the protein, and are therefore selectively neutral, while others may improve enzyme function and be favored by selection.

Originally, evolutionary biologists and geneticists thought genetic diversity in populations would be quite low due to purifying selection [3], but early studies of protein polymorphism revealed unexpected levels of polymorphism at most loci, in a range of organisms including mice, humans, and fruit flies [1-3]; as a result of the high levels of diversity revealed, allozymes were thought to be neutral [4,5]. However, others believed that allozymes would be subject to selection, and subsequent studies have supported selection in many cases [e.g., [4,6]]. Thus, a debate over whether allozymes were neutral or were subject to selection began soon after their discovery, and it has been stated that "few subjects in biology have been more strongly debated than the evolutionary significance of protein polymorphisms" [7].

As enzyme function depends on temperature, pH, substrate concentration, and other environmental factors, some amino acid substitutions will result in an enzyme that functions best under certain conditions, and these may be favored locally by natural selection, depending on environmental factors. In populations inhabiting heterogeneous environments, multiple alleles at one enzyme locus may be maintained by balancing selection [8-10]. Evidence for selection acting upon allozyme loci includes the presence of clines in allozyme allele frequencies [7], correlations between environmental variables and allozyme allele frequencies [11], differences in chemical properties between allozyme alleles [12], and differential performance and/or fitness differences between individuals with different allozyme genotypes [e.g., [5,6,8,13,14]]. These types of evidence are often combined within a system, and several lines of evidence together provide support for the hypothesis that selection is acting on certain allozyme loci (see below).

Another important evolutionary question which is often overlooked is 'why are common alleles common?'. Such common alleles may be prevalent across a wide environmental landscape for many reasons, including ancestral inertia, recent range expansion, genetic drift, purifying selection, or some combination of these or other processes. While it is not always possible to determine the underlying processes that drive or maintain the existence of common allozyme alleles, experiments testing for differential enzyme performance of alleles across a wide-range of environmental conditions can shed light on the possibility of commonness being maintained by natural selection.

Numerous studies providing strong evidence for both neutrality and selection of allozyme loci are found in the literature, and only a few will be detailed here. Cases of neutrality include widespread surveys of allelic variation in white spruce [15], *Peromyscus *mice [16], and several others [4,17]. In contrast, strong evidence for selection has been found for two well-studied loci, phosphoglucose isomerase (*Pgi*; [11,13]) and alcohol dehydrogenase (*Adh*; [4,18]) in a wide range of organisms, and for other loci on a smaller scale [e.g., [7,8]]. Another allozyme locus, isocitrate dehydrogenase (*Idh*), has been studied less than the well-known examples above, but evidence of natural selection acting on this locus has been found across a range of taxa, including bacteria [19,20], plants [21,22], invertebrates [23,24], and vertebrates [25,26]. Isocitrate dehydrogenase is a metabolic enzyme in the Krebs cycle, and its activity is therefore one of several key steps in the generation of ATP from glucose [5]. In the striped ground cricket, *Allonemobius socius*, there is a naturally-occurring cline in *Idh*-1 allele frequencies, hypothesized to have resulted from natural selection [14]. Here, we further test this hypothesis using enzyme kinetics (see below).

**Figure 1:**
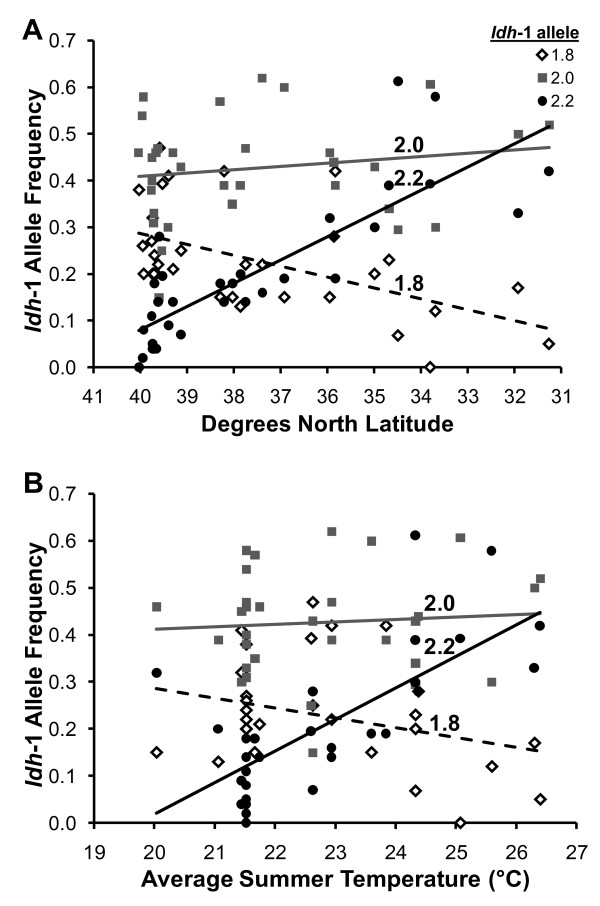
**Geographic variation in allele frequencies at the *Idh*-1 locus in the cricket *Allonemobius socius***. (**A**) Allele frequency data from field-collected populations (data from [14]), represented by individual points; lines are least-squares regressions. The 1.8 allele is symbolized by open diamonds and a dashed line, the 2.0 allele by grey squares and a grey line, and the 2.2 allele by black circles and a black line. (**B**) Relationship between allele frequencies and average summer (June-August) temperature near collection locality.

It has been proposed that four criteria are needed to demonstrate selection on a single allozyme locus [27]. First, populations must contain allelic variation at the locus in question. We have previously demonstrated this to be the case for *Idh*-1 in *A. socius*, as there is significant geographic variation in the allele frequency distributions of two *Idh*-1 alleles (1.8 and 2.2), while a third allele (2.0) is found at a frequency of approximately 50% in most locations (Figure 1A). Next, it must be demonstrated that the observed allelic variation is correlated with an ecological variable (or variables), thus linking natural variation in frequencies to a possible selective force. In *A. socius*, the significant geographic variation in the frequencies of the 1.8 and 2.2 alleles is coupled with significant correlations between allele frequencies and two important environmental variables, temperature (Figure 1B, [14]) and rainfall [14]. Third, there must be phenotypic or fitness consequences to individuals possessing particular alleles in particular environments, providing variation on which selection may act. A previous empirical study of homozygous individuals revealed a significant genotype-by-environment interaction between *Idh*-1 genotype and temperature on fitness in the direction hypothesized based on the environmental correlations [14], providing experimental evidence supporting temperature-driven selection on the *Idh*-1 locus in *A. socius*. The fourth criterion for demonstrating selection on a single enzymatic locus is to show that different allelic variants perform differently under the environmental conditions with which they are correlated (i.e., temperature). Together, these four lines of evidence show that natural variations in allele frequencies are linked to ecologically-relevant differences in enzymatic performance, ultimately leading to differences in organismal fitness on which selection can act [27].

If selection is acting on a locus of interest, then alleles for that locus which occur at a high frequency across a broad geographic range are expected to outperform less common alleles across a wide range of environmental conditions. Additionally, if clinal variation at a locus of interest is driven by selection, then the common allele in a given environment is expected to outperform less common alleles in that same environment. In contrast, if a locus of interest is evolving under drift, then alleles which occur at a relatively high frequency across a broad geographic range are not expected to perform better than other alleles across a wide range of environmental conditions. Moreover, if clinal variation at a locus is a by-product of drift, then there is no expectation that the common allele in a given environment will perform better than other, less common alleles in that environment. These predictions based on drift also hold for a locus of interest that is evolving neutrally but linked to a locus that is under selection. In both cases, common alleles at the locus of interest in a given environment are not expected to perform better than, or increase fitness relative to, other less common alleles.

Here, we use enzyme kinetics techniques to address the fourth criterion for demonstrating selection acting on a locus for *Idh*-1 in the model cricket *A. socius*, and test two specific hypotheses based on the selection vs. drift predictions outlined above: 1) that the 2.0 allele is common across all thermal environments because it performs better than other alleles over a wide range of temperatures, and 2) that the clinal distributions of the 1.8 and 2.2 alleles are due to differences in performance across temperatures, consistent with their geographic distributions. If these two hypotheses are supported, and different alleles perform differently as predicted by their geographic distributions, then there will be strong evidence in support of the fourth criterion required to show selection is acting on the *Idh*-1 locus in *A. socius*.

To further explore the hypothesis that selection has shaped allelic distributions of the *Idh*-1 locus in *A. socius*, we performed enzyme kinetics assays at a range of ecologically-relevant temperatures (ranging from 18–36°C; see Figure 1B) to explore the molecular basis of the GxE interaction between *Idh*-1 genotype and temperature. For all 3 kinetic parameters examined (K_m_, V_max_, and enzyme efficiency), there was a significant GxE between *Idh*-1 genotype and temperature which affected enzyme performance. Additionally, there were significant differences in performance parameters between alleles at both high and low temperatures. Together, these results provide additional evidence that natural selection underlies the naturally-occurring geographic distribution of *Idh*-1 alleles in *A. socius*.

## Methods

### Study System

Crickets of the genus *Allonemobius *range from southern Canada to the southern United States, primarily in the East, and are abundant in appropriate habitat throughout their range. Due to their large distribution, abundance in the field, and ease of laboratory maintenance, members of the *A. socius *complex have been used as a model system for several aspects of evolutionary biology, including studies of speciation and hybrid zone dynamics [28-30], *Wolbachia *[31,32], life-history evolution [14,33-36], and morphological variation [33,37-39]. Additionally, given their naturally widespread distribution, the *A. socius *complex is an ideal model system for studying geographic variation in life-history traits and genetic diversity. Within the *A. socius *complex, clines have been found in ovipositor length [37], diapause occurrence [33,34,40], allozyme alleles [14,28,30], and nuclear and mitochondrial markers [31].

Members of the *A. socius *complex are morphologically cryptic, and species were originally discovered and described using allozymes [28,41,42]; therefore, much data about the geographic distribution of allozyme alleles are readily available. One locus in particular, isocitrate dehydrogenase (*Idh*-1), is strongly clinal within *A. socius*, with the 1.8 allele being common in the north and east and the 2.2 allele being common to the south and west (Figure 1A; [14]).

The geographic distributions of these alleles are also correlated with two important environmental variables, temperature (Figure 1B) and rainfall, with 1.8 at highest frequency in cooler, drier locations while 2.2 is associated with hotter, wetter locations [14]. A third allele, 2.0, is found at intermediate frequencies in most locations (~50%; Figure 1A) and not significantly distributed in relation to geography or climate [14]. In a previous study, we found a significant interaction between *Idh*-1 genotype and temperature on fitness, such that individuals homozygous for the 1.8 allele laid more eggs at a cool temperature relative to the two faster alleles, and individuals homozygous for the two faster alleles laid more eggs at a warm temperature than 1.8 individuals [14]. However, the molecular basis for this GxE was not examined. Here we use enzyme kinetics to assay differences in performance between these 3 alleles across a range of ecologically-relevant temperatures. Temperatures chosen ranged from 18–36°C, and were chosen to reflect temperatures experienced by populations in the field across the species' range (see Figure 1B).

### Kinetic Parameters and Calculations

**Figure 2:**
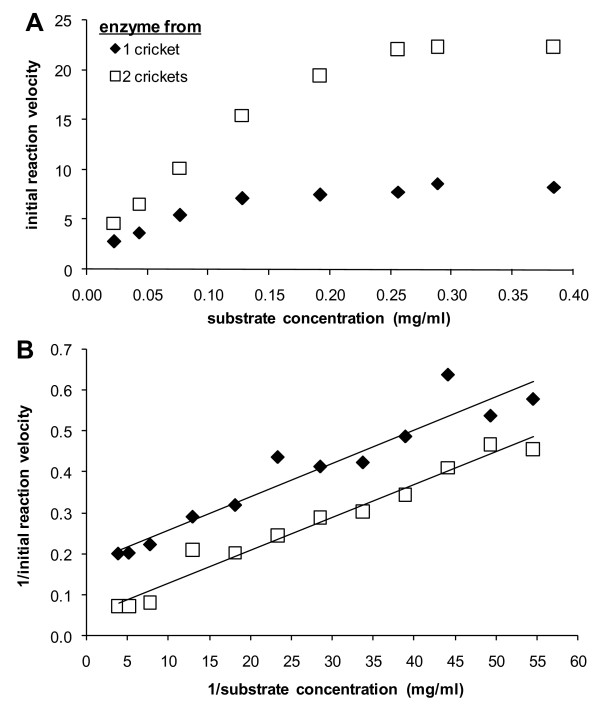
**Example enzyme kinetics data**. Experimental *Idh*-1 kinetic data using homogenates from 1 cricket (2 legs; solid diamonds) and 2 crickets (4 legs; open squares). (**A**) Initial reaction velocity as a function of substrate concentration, showing the expected hyperbolic relationship. Reaction velocity increases linearly until approaching the saturation point. (**B**) Lineweaver-Burk plot, the double-reciprocal method for calculating K_m_, the Michaelis constant, and V_max_, the maximum reaction velocity, from kinetic assay data.

The initial velocity of an enzyme-catalyzed reaction depends on the initial substrate concentration; this relationship is typically hyperbolic, with a linear increase at lower concentrations until the reaction approaches saturation, at which point further increases in substrate will not increase reaction velocity (see Figure 2A). Two important parameters are typically calculated using kinetic assay data: V_max_, the initial reaction velocity at saturated substrate concentration, and the Michaelis constant, K_m_, which is a measure of the affinity of the enzyme for the substrate and the rate at which the substrate is converted to product [43]. These parameters are obtained through a double-reciprocal plot of velocity against substrate concentration for the linear portion of the original curve (see Figure 2B). First, least-squares regression is performed on the double-reciprocal data and the slope and y-intercept calculated. V_max _is then calculated from the regression equation, using the formula: V_max _= 1/y-intercept [43]. Next, K_m _is obtained from the equation: K_m _= V_max _*slope [43].

Because the y-intercept changes with enzyme concentration (Figure 2B), K_m _and V_max _are also influenced by the initial enzyme concentration, such that the values of both parameters increase when enzyme concentration increases (Table 1). However, the slope of the least-squares line is not dependent on the initial enzyme concentration (Figure 2B, Table 1), and is equal to K_m_/V_max_, a measure of enzyme efficiency. As V_max _is a measure of reaction velocity, a relatively higher value of V_max _means the reaction can progress faster, and therefore higher values of V_max _indicate better enzymatic performance. Conversely, K_m _contains a measure of enzyme-substrate affinity, and is the amount of substrate needed to achieve one-half V_max_. Therefore, an enzyme with a lower K_m _needs less substrate to achieve a given rate than one with a higher K_m_. Lastly, because enzyme efficiency (as defined above) is calculated from reciprocal plots, a smaller value of this parameter (either due to a lower K_m _and/or a higher V_max_) means the enzyme is more efficient than a higher value. Here we report all 3 kinetic parameters (efficiency, K_m_, and V_max_), but note that K_m _and V_max _may be influenced by variation in amount of enzyme present in each individual. Specifically, differences in these two parameters when comparing two individuals could be due to differences in the relative amounts of enzyme in individuals (see Table 1) or real differences in performance between individuals for these two parameters.

**Table 1:** Estimation of 3 enzyme kinetic parameters from data presented in Fig. 2.

parameter	1 cricket	2 crickets	Ratio (2/1)
Efficiency	0.0081	0.0080	0.9877
V_max_	5.6180	20.0803	3.5743
K_m_	0.0455	0.1606	3.5302

This table demonstrates that kinetic efficiency is nearly equal with approximately double the amount of enzyme, while both K_m _and V_max _increase by a factor of approximately 3.5.

### Experimental Methods

#### Experimental Animals

To further detail the relationship between temperature and enzyme activity in *A. socius*, we conducted an enzyme kinetics experiment using tissue homogenates derived from laboratory-raised *Idh*-1 homozygotes. Juvenile crickets were collected in July 2006 from a field population in western North Carolina (35.199°N, 81.371°W), an area known to have all 3 alleles at roughly equal frequencies (see Figure 1 in [14]). Field-collected juveniles were raised to adulthood at 27°C in sex-specific cages to prevent mating. Adults were individually genotyped for malate dehydrogenase (*Mdh*-1; diagnostic of the *A. socius *complex relative to other species in the genus) and isocitrate dehydrogenase to screen for individuals homozygous at the *Idh*-1 locus. Allozyme electrophoresis and staining was performed using standard methods for *Allonemobius *[28].

Individuals homozygous for each of the 3 alleles (1.8, 2.0, and 2.2) were placed in genotype-specific mating cages and allowed to mate for 2 weeks, producing homozygous offspring. Offspring were raised to adulthood at 27°C and ~10 individuals per genotype were randomly chosen and frozen at -80°C. For these individuals, *Idh*-1 genotype was confirmed with allozyme electrophoresis on head tissue as above. Enzyme homogenates from 3 individuals of each genotype were generated by homogenizing both rear legs in 25 μl 0.2 M tris-citrate buffer, pH 8.0 [28], centrifuging for 2 minutes at 10,000 × *g*, and removing the supernatant for use in enzyme kinetics assays.

#### Kinetics Assays

Enzyme kinetics assays were performed in 96-well microplates using a temperature-controlled microplate reader (Bio-Tek Instruments, Winooski, VT), similar to the procedure described by [44]. A pilot study was conducted using a wide range of substrate concentrations and standard *Idh *staining media optimized for *Allonemobius *([28]; 0.2 M tris-citrate buffer, pH 8.0 with 1 mg/ml MgCl_2_, 0.2 mg/ml NADP, 0.2 mg/ml NBT, and 0.04 mg/ml PMS; all reagents from Sigma-Aldrich) to determine the range of concentrations which produced a linear relationship between initial reaction velocity and substrate concentration (see Figure 2A; [43]). This staining solution produces a purple color as the reaction progresses, and absorbance was read at 595 nm using the microplate reader. Although this assay measures combined activity for *Idh*-1 and *Idh*-2, the *Idh*-2 locus is monomorphic in *A. socius *(and all species of *Allonemobius*; [14,29,30,42]); therefore, all differences observed between *Idh*-1 genotypes should result from variation in performance of *Idh*-1 alleles. From these preliminary data, four concentrations of isocitric acid were used for further assays (0.077, 0.055, 0.043, and 0.035 mg/ml).

Next, assays were performed on 3 individuals per genotype (i.e., 1.8, 2.0, and 2.2 homozygotes) at 7 ecologically-relevant temperatures (18, 21, 24, 27, 30, 33, and 36°C; see Figure 1B). For each individual, at each temperature and substrate concentration, a 1 μl aliquot of enzyme homogenate was added to 25 μl of staining media and used in an assay following the protocol outlined above. All samples for each temperature were conducted simultaneously with absorbance readings taken for every 15 sec for 20 minutes (yielding 80 absorbance readings per aliquot).

Using standard Michaelis-Menten kinetics [e.g., [43,45]] implemented by the program KC3 (Bio-Tek Instruments), initial reaction velocity of the enzymatic conversion of isocitrate to α-ketoglutarate was calculated using the linear portion of the curve (approximately 5–15 minutes). For each individual at each assay temperature, the increase in velocity with increasing isocitric acid concentration was plotted and slopes calculated (see *Kinetic Parameters and Calculations *above; Figure 2B), yielding a measure of enzyme efficiency – i.e., the inverse of the change in the rate of the reaction with increasing substrate concentration. K_m_, and V_max _were also calculated from the assay data using standard methods as described above.

### Statistical Analyses

Kinetic data were analyzed using repeated-measures analysis of variance (ANOVA) with *Idh*-1 genotype as a between-subjects factor and assay temperature and the interaction of temperature and *Idh*-1 genotype as within-subjects factors. Enzymatic efficiency, K_m_, and V_max _were analyzed using separate ANOVA's. Post-hoc ANOVA's were then used to test for significant differences between alleles at each temperature and groupings assigned using Ryan-Einot-Gabriel-Welsch (REGWQ) multiple-range tests [46]. All statistical analyses were performed with SAS Learning Edition 4.1 [46]. Statistics were considered significant at *P *< 0.05.

## Results

**Figure 3:**
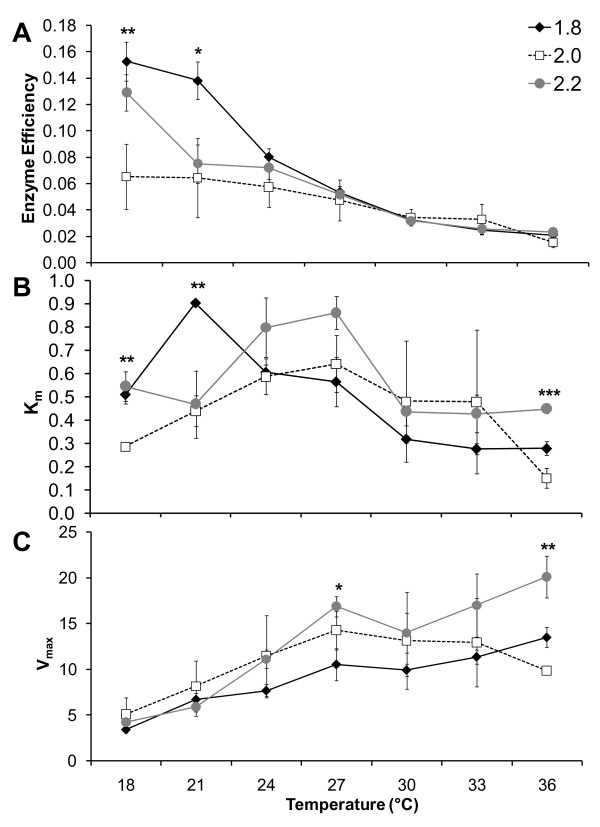
**Kinetic performance across a temperature gradient of 3 *Idh*-1 alleles in the cricket *Allonemobius socius***. Enzyme efficiency (**A**) was measured as the increase in reaction velocity with increasing substrate concentration (see Methods). K_m _(**B**) and V_max _(**C**) were calculated using standard Michaelis-Menten methods. Means ± SE are given (*n *= 3 individuals per genotype). Individual ANOVA's were used to test for differences between genotypes at each temperature and significant differences indicated (*P < 0.1, **P < 0.05, ***P < 0.01).

There was a significant interaction between *Idh*-1 genotype and assay temperature that affected enzyme efficiency, measured as the increase in velocity with increased substrate concentration (Table 2). Because of the inverse relationships plotted by this method, a lower value indicates greater efficiency. Enzyme efficiency was significantly different between alleles at the two lowest temperatures tested (18 and 21°C; Figure 3A), while there was no difference at the higher temperatures tested (24–36°C; Figure 3A). At 18°C, 2.0 individuals outperformed 1.8 and 2.2 individuals, which were not significantly different from each other, while at 21°C, 2.0 and 2.2 individuals were not significantly different and more efficient than 1.8 individuals (Figure 3A).

**Table 2:** Repeated-measures ANOVA on kinetic efficiency (see Methods) across a temperature gradient.

Between Subjects				
Source	df	MS	*F*	*P*

Genotype	2	0.00303	5.07	0.0627
Error	5	0.00060		

Within Subjects				

Source	df	MS	*F*	*P*

Temperature	6	0.00996	39.89	< 0.0001
Genotype × Temperature	12	0.00108	4.31	0.0009
Error (Temperature)	30	0.00025		

Within-subject *P*-values are with Huynh-Feldt corrections.

There was also a significant GxE between *Idh*-1 genotype and assay temperature for K_m_, the Michaelis constant (Table 3). K_m _is a measure of the binding affinity between the enzyme and the substrate, and a lower value of K_m _indicates a higher affinity. There were significant differences in K_m _between alleles at 3 temperatures (the two lowest temperatures, 18 and 21°C, and the highest temperature, 36°C; Figure 3B). Similar to the results for efficiency (see above), at 18°C, 2.0 individuals had lower K_m _than 1.8 and 2.2 individuals, which were not different from each other, while at 21°C, 2.0 and 2.2 individuals were not significantly different from each other and had lower K_m _than 1.8 individuals (Figure 3B). At 36°C, individuals of all three alleles were significantly different from each other, with 2.0 having the lowest K_m_, followed 1.8 and 2.2 (Figure 3B).

**Table 3:** Repeated-measures ANOVA on K_m _across a temperature gradient.

Between Subjects				
Source	df	MS	*F*	*P*

Genotype	2	0.07596	2.94	0.1431
Error	5	0.02582		

Within Subjects				

Source	df	MS	*F*	*P*

Temperature	6	0.17803	7.79	0.0010
Genotype × Temperature	12	0.06115	2.67	0.0426
Error (Temperature)	30	0.02286		

Within-subject *P*-values are with Huynh-Feldt corrections.

Lastly, the maximum reaction velocity, V_max_, was affected by a significant GxE between *Idh*-1 genotype and assay temperature (Table 4). At 36°C, 2.2 had a significantly higher V_max _than 1.8 and 2.0, which were not significantly different (Figure 3C). These data indicate that the 2.2 allele encodes an enzyme with the highest velocity but also the highest K_m _at 36°C, an expected tradeoff between velocity and affinity [5].

**Table 4:** Repeated-measures ANOVA on V_max _across a temperature gradient.

Between Subjects				
Source	df	MS	*F*	*P*

Genotype	2	73.7718	1.67	0.2787
Error	5	44.2408		

Within Subjects				

Source	df	MS	*F*	*P*

Temperature	6	119.7032	25.46	< 0.0001
Genotype × Temperature	12	13.5814	2.89	0.0133
Error (Temperature)	30	4.7018		

Within-subject *P*-values are with Huynh-Feldt corrections.

## Discussion

Previous studies have shown that the effect of temperature on enzyme performance can affect many aspects of organismal fitness, including growth rate and size at maturity [47]. Thus, based on previous geographic and empirical data for *A. socius*, we hypothesized that the observed distributions of *Idh-1 *alleles, including the high frequency of the 2.0 allele and the cline in the frequency of the 2.2 allele, had resulted from natural selection on differential enzymatic performance across thermal environments. By conducting kinetics assays, we found a significant GxE between *Idh*-1 genotype and temperature on 3 measures of enzyme kinetic performance in *A. socius*. Additionally, we found significant differences in performance among alleles at the two lowest and one highest temperatures.

Specifically, at 18°C, 2.0 was more efficient because of higher substrate affinity (lower K_m_) of the Idh enzyme than the 1.8 and 2.2 alleles. At 21°C, the 2.0 and 2.2 alleles both had higher substrate affinities that that of 1.8 for those two parameters; there was no significant difference among alleles in V_max _at low temperatures. At 36°C, there was no significant difference in overall efficiency between alleles. However, K_m _and V_max _were significantly higher for the 2.2 allele. K_m _and V_max _for 2.0 were lower than the other alleles at this temperature, while the 1.8 allele had intermediate values for K_m _and V_max _when compared to the other two. Overall, we found that K_m _and V_max _varied at critical temperatures for all 3 alleles, in contrast to the results of Johns and Somero [48], who found that a cold-adapted allele of lactate dehydrogenase (*Ldh*-4) had a higher K_m _than warm-adapted alleles across all assay temperatures in Pacific damselfishes.

The kinetics results, in general, are consistent with hypothesized results based on the naturally-occurring distribution of *Idh-1 *allele frequencies and previous fitness data [14]. Specifically, the 2.0 allele was the most efficient allele at lower temperatures (≤ 27°C) and equivalent in efficiency to the other two alleles at higher temperatures (> 27°C; Figure 3A); these data indicate that the 2.0 allele performs well across the widest range of temperatures, and it is therefore not unexpected that it occurs at approximately 50% frequency in all populations in the eastern United States (Figure 1A; [14]). Conversely, the 2.2 allele had the highest maximum reaction velocity (V_max_) at higher temperatures (≥ 27°C); this allele occurs at approximately equal frequency with the 2.0 allele at lower latitudes, but is not as common to the north (Figure 1A). However, it is somewhat surprising that the 1.8 allele did not perform better at lower temperatures (18 and 21°C), given the earlier fitness data and its geographic distribution (Figure 1; [14]). At the lowest assay temperature (18°C), the 1.8 allele was equivalent to the 2.2 allele in both efficiency and substrate affinity, while the V_max _of all 3 alleles were not significantly different. Thus, we hypothesize that the increased frequency of the 1.8 allele in northern latitudes may be more reflective of processes such as genetic drift, rather than a strict by-product of natural selection. In all, our findings support the hypotheses that 1) the 2.0 allele is common across all thermal environments due to superior performance, for at least some kinetic parameters, relative to the other two alleles at many different temperatures, and 2) the clinal distribution of the 2.2 allele is driven by selection on thermal performance. Combining these data with an earlier study on geographic variation, environmental correlates, and fitness data [14], *Idh*-1 in *A. socius *now meets the criteria described by Mitton [27] and others (e.g, [5-8,11-13]) for demonstrating selection on a single enzyme locus.

In general, enzymatic performance depends on temperature and typically decreases at high temperatures due to degradation and inactivation of enzyme molecules [5,49-52]. Interestingly, we did not observe a decrease in performance, even in our highest temperature assays (Figure 3). Therefore, the temperature range over which we conducted our assays appears not to have exceeded the thermostability threshold of the isocitrate dehydrogenase enzyme over the time period of the assay, which is not surprising given that temperatures were chosen to reflect the natural conditions experienced by field populations.

At extreme reaction temperatures (cold or hot), researchers typically observe a tradeoff between K_m _and V_max_. Although an enzyme may tightly bind a substrate, the rate at which the substrate is converted to product may be lower [5,13,53-56]. Our data appear to show this tradeoff at our highest assay temperature (36°C), as the 2.2 allele has both the highest K_m _and V_max_, while 2.0 has the lowest (Figure 3). These data may be influenced by overall differences in amounts of Idh enzyme produced by individuals of different genotypes. However, if differences in performance of alleles at 36°C were due to differences in Idh concentrations among the samples, we would expect alleles to show similar patterns of performance for these two parameters across all temperatures, not just 36°C. Overall, we found that at low temperatures all three alleles have a similar V_max _but significant differences in efficiency and K_m_, while at high temperatures all three alleles have the same efficiency but significant differences in K_m _and V_max _(see Figure 3). These findings point to a clear performance tradeoff, in that a single allele cannot perform optimally for all measures of kinetic performance across all temperatures.

When one genotype produces different phenotypes over a range of environmental conditions, the relationship between the phenotype produced and the environment is known as a reaction norm [57]. Similarly, a genotype-by-environment interaction (GxE) occurs when different genotypes produce different reaction norms across the same environmental conditions. Reaction norms and GxE interactions are thought to be adaptive for species living in temporally-variable environments or with wide geographic ranges, as populations may experience different environmental conditions across the species' range or during the year. Such environmental variation can lead to balancing selection on alternative alleles and/or the evolution of phenotypic plasticity. Given GxE and spatially- or temporally-variable environments across a species' range, balancing selection is predicted to maintain genetic diversity in natural populations [5,9,10,58]. It appears that all 3 alleles are being maintained in *A. socius*, possibly due to differences in GxE across temperatures.

There are a few alternative explanations for the allele-frequency distributions observed in natural populations of this species. For example, it is also possible that *Idh*-1 is linked to another locus which is under temperature-driven selection, and that the allele frequencies observed in nature reflect this linkage disequilibrium [5,27]. However, our kinetics assays were designed to specifically measure Idh activity, and we observed significant GxE. Alternatively, there could be other genes in the Krebs cycle or other metabolic pathways which are affected by *Idh*-1 activity and that selection is acting on the pathway as a whole [5]. To further test the hypothesis that natural selection (and/or genetic drift) is acting specifically on the *Idh*-1 locus in *A. socius*, we are currently sequencing the protein-coding region for all alleles from multiple individuals spanning the geographic range of this species.

The link between allozyme allele frequencies, differential thermal performance of alleles, and natural environmental gradients has been shown in several other recent studies. For example, Piccino et al. [58] found significant differences in phosophoglucomutase (*Pgm*-1) allele frequencies between populations of the polychaete *Alvinella pompejana *which inhabited either newly-created or older hydrothermal vents. The enzyme allele at highest frequency in populations living near recently-established, warmer vents was both more thermostable and had higher activity at warmer temperatures than the allele found in populations dwelling in older, cooler vents [59]. Similar results have been found for *Ldh *in species of Pacific damselfish of the genera *Chromis *[48] and *Sphyraena *[60] inhabiting different thermal regimes. Similarly, clines in allele frequencies of *Pgi*-1 in the leaf beetle *Chrysomela aeneicollis *were linked to enzyme kinetic performance across ecologically-relevant temperatures, providing strong evidence of temperature-driven selection [61]. Together, these and other studies indicate that some allozyme loci are not neutral markers of diversity, but rather that the geographic distributions of alleles can be a consequence of environmental conditions and the differential performance of alleles in those environments. Thus, common alleles may be common due to their enhanced performance relative to other, less-common alleles, while clinal distributions can be attributed to either selection or drift across thermal gradients. The distribution of *Idh*-1 alleles in *A. socius *appears to be another example of this phenomenon, as we are now able to link a GxE between genotype and temperature on enzyme kinetic performance to the natural distribution of alleles in this species.

## Conclusion

Clines in allele frequencies within a species can be caused by natural selection on allele variants along an environmental gradient or by genetic drift across the species' range. Similarly, common alleles may be common due to superior fitness or to genetic drift. Previously, we hypothesized that natural selection may be maintaining a natural cline in *Idh*-1 allele frequencies (1.8, 2.0, and 2.2) in the cricket *A. socius*, due to both correlations between allele frequencies and environmental conditions and fitness differences between homozygotes of the various alleles across temperatures [14]. Using enzyme kinetics to further dissect the GxE between *Idh*-1 genotype and temperature at the molecular level, we found significant differences in enzymatic performance between alleles across temperatures. These data suggest that 1) natural selection is maintaining the cline in frequency of the 2.2 allele, 2) the 2.0 allele is common across a wide geographic range because it performs well across a broad range of temperatures, and 3) drift may be acting on the 1.8 allele. Together, our data indicate that natural selection is acting on the *Idh*-1 locus in *A. socius*. Although these enzymatic performance data point to selection maintaining the high frequencies of the 2.0 and 2.2 alleles in given environments, we still have not assessed the molecular signature of positive or balancing selection on the *Idh*-1 locus. To fill this gap, we are currently sequencing *Idh*-1 alleles from populations that span the geographic range of *A. socius *and will be assessing patterns of synonymous and nonsynonymous changes to identify any allele-specific signatures of molecular evolution.

## Abbreviations

*Idh*: isocitrate dehydrogenase; GxE: genotype-by-environment interaction; K_m_: the Michaelis constant, a measure of affinity; V_max_: the maximum reaction velocity at saturating substrate concentration.

## Authors' contributions

DLH co-conceived of the study, carried out laboratory experiments, co-analyzed the data, and drafted the manuscript. BO helped develop laboratory protocols, supervised laboratory experiments, and contributed to writing of the manuscript. JLM co-conceived the study, co-analyzed the data, and contributed to writing of the manuscript. All authors read and approved the final manuscript.
